# Everybody's Page

**Published:** 1890-10-04

**Authors:** 


### Everybody's Page.

Only one death occurred from small-pox in Holland last year.

This is a warning to householders.

His Honour: "What made you steal this gentleman's door-mat?"

Prisoner: "Sure, your honour, it said 'Welcome' on it in letters as long as your ar-r-m!"

The inhabitants of New York are much disgusted with the result of the recent census, so a new count is to take place. The citizens have set their hearts on having a population of 1,700,000, and it will be well for Mayor Grant if the second count brings the numbers up to the required standard.

Of towns in England which empty their sewage into the river, Bath is a very fine specimen. The river, when at all empty shows a plentiful supply of slimy, black mud on the banks, and the smells there, are, we should say, unequalled. The sewers are placed, we believe below the summer level of the river. This is precautionary but not satisfactory, judging by the odours we mention.

Professor Garrison, of Chicago, is of opinion that the crumpled up form of the human ear was originally caused, and still remains the same, on account of the habit of lying on the side of the head, the great weight of the brain rendering this the most comfortable position. Whether we should have looked better with ears erect it is unnecessary to discuss. Nature evidently decided that for man great brains were of more importance than perfect acoustic faculties.

Vegetarianism is looking up: "Three Brothers" write to the *Daily News*, saying that all they need to enable them to become vegetarians is a cookery book on that form of diet. Surely this is not remissness on the part of the oracles of vegetarianism; we should have expected they would have ssued scores of books to aid the young beginner. Can it be that it has only been the want of a cookery book which has up to now deterred the public from adopting this highly economical form of food?

It is said that were it not for its great weight peat would play a considerable part in the preservation of alimentary substances, and consequently facilitate the exportation of ood to great distances. Fish was preserved by Dr. Fürst for eighteen days in dried peat, and at the end of that period was in perfect condition. Sausages and meat have been kept in it successfully, and at the Magdeburg Exhibition in 1888 a quarter of raw pork was shown which had been preserved in peat since the summer of 1887; the meat was dried up, but there was not the slightest smell or sign of decomposition. M. Louis Passy has been drawing the attention of the Société Nationale d'Agriculture to this interesting subject.

The *Queen* pays a tribute to the merits of tricycling for ladies, saying that, moderately indulged in, it is an excellent form of exercise for womankind. "We cannot," says the journal, "legislate for exceptional cases, or for excessive and violent use; you may overdo tricycling as you may overdo everything else." But this is not the only question to be considered; it is generally conceded that treadle sewing machines are not the very best things for even healthy women to use, and what holds good of them holds good of the tricycle treadles; also there is the question of the saddle, which may suffice very well for a woman of light weight, but which is certainly not support enough for a heavy person.

Eight women have been appointed factory inspectors in the State of New York.

An American custom has just been adopted at the Ambigu Theatre in Paris—that of placing between each two chairs an opera glass, which is released for use by dropping a half franc into an automatic case. It is to be hoped that the glasses provided are of better quality than those usually supplied in English theatres for double the price.

The Manchester School Board, at their first meeting since Miss Becker's death, passed a resolution that her former colleagues on the Board will ever gratefully remember the earnestness of purpose, constant zeal, and unwearied energy in all matters relating to public elementary education, and the amelioration of the condition of the working classes which Miss Becker invariably showed. Miss Becker's place has been filled by Mrs. C. P. Scott, an unsectarian member of the School Board.

The year 1724 is said to be about the period when gin-drinking began to take such hold upon the masses in England. In those days an alluring poster fastened to the door of the gin cellars announced that for a penny a man could satisfy his thirst, and for twopence he could drink gin to satiety. It is said that from about this period the number of liver complaints and dropsy increased at an alarming rate. Gin drinking is said to have decreased of late years; it has certainly done so among the upper classes, but whether it has done so among the lower classes seems questionable.

At the recent visit paid by several members of the Association of Public Sanitary Inspectors to the crematorium at Woking, it appears that some and the only objections which were raised to the practice of cremation were on the ground of sentiment. The feeling in its favour on sanitary grounds is, we are glad to say, rapidly deepening into conviction, and the best proof that adverse sentiment, which seems now to be the only great obstacle to its more general introduction, is on the wane, is the fact that, of the 138 cremations which have taken place during the thirteen years of the Cremation Society's existence, 38 have taken place during the past nine months. Such a rapid rate of increase speaks volumes.

Dr. Drysdale, lecturing on the "Sewage of Towns" on Friday night of last week, declared that the only system of treating the sewage of large cities was that used in Paris, Berlin, Croydon (and some other places on a much smaller scale,) namely the agricultural application of the sewage on suitable soils. The lecturer had not a good word for London, which city, he said, offered a bad example to other cities by turning the Thames into a vast sewer, or by inadequately dealing with the solid matter. He quoted the plain of Gennevilliers, where 1,500 acres are cultivated by small proprietors who use 20,000,000 tons per annum of the Paris sewage to irrigate their farms. Berlin has 19,000 acres under cultivation as sewage farms on which no case of typhoid has occurred during the year, not to mention that two convalescent hospitals have been established on the farms themselves, which is, what we think to be rather stretching the point. At both Paris and Berlin the effluents are perfectly clear. The Englishman, however, is conservative. The Thames is ugly, useful, and smells, but so the Englishman prefers it; he might try an experimental farm, but he will take a long time to make up his mind whether he will or no. Meanwhile he is happy contemplating the muddy stream as it is.

